# Environmental Enrichment Attenuated Sevoflurane-Induced Neurotoxicity through the PPAR-*γ* Signaling Pathway

**DOI:** 10.1155/2015/107149

**Published:** 2015-07-06

**Authors:** Yupeng Zhao, Kaizheng Chen, Xia Shen

**Affiliations:** ^1^Department of Anesthesiology, Shanghai East Hospital, Tongji University School of Medicine, No. 150, Jimo Road, Shanghai 200120, China; ^2^Department of Anesthesiology, The Eye, Ear, Nose and Throat Hospital of Fudan University, Shanghai Medical College of Fudan University, Shanghai 200031, China

## Abstract

Sevoflurane is the most widely used inhaled anesthetic. Environmental enrichment (EE) can reverse sevoflurane-induced learning and memory impairment in young mice. However, the mechanism by which EE elicits this effect is unclear. The peroxisome proliferator-activated receptor (PPAR) regulatory pathway plays a critical role in the regulation of inflammation in central nervous system diseases. In this study, we investigated whether EE attenuates sevoflurane-induced learning and memory disability via the PPAR signaling pathway. Six-day-old mice were treated with 3% sevoflurane for 2 hours daily from postnatal day 6 (P6) to P8. Then, the mice were treated with EE. The effects of sevoflurane on learning and memory function, PPAR-*γ* expression in the brain, and the numbers of terminal deoxynucleotidyl transferase dUTP nick end labeling-positive cells and 5-bromodeoxyuridine-positive cells in the hippocampus were determined. Sevoflurane induced neuronal apoptosis and neurogenesis inhibition, which may impair learning and memory in young mice. Furthermore, sevoflurane downregulated PPAR-*γ* expression. Both EE and the PPAR-*γ* agonist, rosiglitazone, attenuated sevoflurane-induced neuronal apoptosis, neurogenesis inhibition, and learning and memory impairment. Our findings suggest that EE ameliorated sevoflurane-induced neurotoxicity and learning and memory impairment through the PPAR-*γ* signaling pathway. PPAR-*γ* may be a potential therapeutic target for preventing or treating sevoflurane-induced neurotoxicity.

## 1. Introduction

Pediatric patients who undergo multiple surgeries also require multiple exposures to general anesthesia. Currently, sevoflurane is the most widely used inhaled anesthetic for general anesthesia in children. Recent studies showed that children with multiple exposures to general anesthesia and surgery at an early age may develop learning and memory disabilities [1, 2]. Sevoflurane has been shown to inhibit the proliferation of neural progenitor cells, decrease the self-renewal capacity of neural stem cells, and induce neuroinflammation in microglial cells in mice [3–6]. Moreover, results from animal studies showed that multiple exposures of sevoflurane may induce neuroinflammation, neuronal apoptosis, and neurogenesis inhibition in the brain tissues of 6-day-old fetal mice. Learning and memory of these mice were subsequently impaired after 3 weeks [7]. Therefore, sevoflurane-induced neurotoxicity in the developing brain is drawing more attention in the context of children who are exposed to inhalational general anesthetics for surgery.

Environmental enrichment (EE) is the stimulation of the brain by its physical and social surroundings. Previous research on animals has demonstrated that EE can play a role in the treatment and recovery of numerous brain-related disorders, such as Alzheimer's disease (AD) and aging-related brain dysfunction, whereas a lack of stimulation might impair cognitive development [8, 9]. These studies suggested that EE might lead to a greater level of cognitive reserve, thus increasing the brain's resilience to conditions, such as aging and dementia [10]. Moreover, research on humans suggested that the lack of stimulation could delay and impair cognitive development [9]. People who attained and engaged in higher levels of education participated in more challenging and cognitively stimulating activities and had greater cognitive reserve [8, 11]. Furthermore, EE has been shown to ameliorate sevoflurane-induced learning and memory impairment [7, 12]. However, the mechanisms by which EE elicits its effects are unclear.

Peroxisome proliferator-activated receptors (PPARs) are members of the nuclear hormone receptor family of ligand-activated transcription factors. There are three PPAR subtypes: PPAR*α*, PPAR*β*/*δ*, and PPAR*γ*. PPAR*γ* has the ability to modulate inflammatory responses and cell survival [13, 14]. Several studies have shown that PPAR*γ* agonists can improve cognitive performance in mouse models of AD [15]. The PPAR-*γ* agonist, rosiglitazone (RSG), is a Food and Drug Administration- (FDA-) approved drug that has been used in the clinical setting to treat diabetes. RSG can cross the blood-brain barrier and induce mitochondrial biogenesis in the mouse brain. It has also been shown to enhance cognition in AD mice through the hippocampal PPAR-*γ* signaling pathway [16]. PPAR-*γ* agonists, including RSG and pioglitazone, can regulate inflammatory processes in the central nervous system and have neuroprotective effects against neurological and neurodegenerative disorders [17].

In this study, we used 6-day-old mice to investigate the mechanism by which EE elicits its effects on sevoflurane-induced learning and memory impairment. Our results showed that both RSG and EE attenuated sevoflurane-induced neurotoxicity. This suggests that EE, at least in part, ameliorates sevoflurane-induced learning and memory impairment through the PPAR*γ* signaling pathway.

## 2. Methods and Materials

### 2.1. Animals Anesthesia and Treatment

Six-day-old C57/BL wild-type male mice were obtained from the specific pathogen-free animal center at Shanghai East Hospital, which is affiliated to Tongji University. All animal experiments were approved by the Animal Care Committee of Shanghai East Hospital-Tongji University.

Mice were treated with 3% sevoflurane (Abbott, Japan) from P6 to P8, as described in our previous study [7]. Specifically, the anesthetic (sevoflurane) was administered with 60% oxygen (balanced with nitrogen), as performed in the previous study [7]. Control groups received 60% oxygen at an identical flow rate in similar chambers. The chamber was located in 37°C thermostat water bath to maintain body temperature of the six-day-old mice. The mice breathed spontaneously in the chamber, and the concentrations of sevoflurane and oxygen were measured continuously. Two hours later, sevoflurane was discontinued and mice were allowed to wake up. RSG (R2408; Sigma-Aldrich, MO, US) was prepared fresh daily with sterile saline to the concentration of 0.1 mg/mL. RSG (3 mg/kg) [15] was administrated intraperitoneally 2 h prior to sevoflurane anesthesia. All treatments were administered intraperitoneally at 2 h prior to sevoflurane anesthesia. P6 mice were intraperitoneally administered 100 mg/kg BrdU at 2 h prior to sevoflurane anesthesia. After EE treatment, P30 mice were administered intraperitoneal injections of 50 mg/kg BrdU (Sigma-Aldrich) for 2 times in 1 day. Behavioral test was performed from P30 to P34 by Morris water maze (MWM); the endpoints of MWM included escape latency from P30 to P34 and platform crossing times at P34. In addition, we used the pups but not litters for this study. Only if the pups died during anesthesia we do not cull any pups. Overall, the mortality rate of mice during anesthesia was less than 1%.

### 2.2. Environmental Enrichment

EE was established, as previously described [7]. EE was created in a large cage (70 × 70 × 46 cm) that included five or six toys (e.g., wheels, ladders, and small mazes). The mice were put in the EE every day for 2 h from P8 to P30. The objects were changed two to three times per week to provide a novel and challenging environment.

### 2.3. Morris Water Maze Test

The MWM test was conducted in a circular tank (diameter: 1.8 m, height: 60 cm) that was filled with opaque water. A platform (11 × 11 cm) was submerged 1.0 cm below the water's surface in the center of the target quadrant. Water was kept at 20°C and opacified with titanium dioxide. The mice were tested in the Morris water maze (MWM) four times per day for 5 days (from P30 to P34). Each mouse was placed in the pool to search for the platform. The starting points were random for each mouse. Mice that found the platform were allowed to stay on it for 15 s. If a mouse did not find the platform within a 90 s period, it was gently guided to the platform and allowed to stay on it for 15 s. A video tracking system recorded the swimming motions of the animals, and the data were analyzed using motion-detection software for the MWM (Institute of Materia Medica, Chinese Academy of Medical Sciences and Peking Union Medical College, Beijing, China). At the end of the reference training (P34), the platform was removed from the pool and the mouse was placed in the opposite quadrant. Each mouse was allowed to swim for 60 s, and the number of times the mouse swam across the platform area was recorded (platform crossing times). Mouse body temperature was maintained by active heating. Specifically, after every trial, each mouse was placed in a holding cage under a heat lamp for 1-2 min to dry before being returned to its regular cage.

### 2.4. Brain Tissue Harvest and Protein Level Quantification

Different groups of mice under the control, anesthesia conditions, and EE were used for biochemistry studies. Immediately after the anesthesia (P8) or EE (P30), the mice were killed by decapitation (for western blot analysis) or transcardial perfusion for paraffin section cutting and immunohistochemistry studies. Separate groups of mice were used for the western blot analysis and the immunohistochemistry studies. For the Western blot analysis, the harvested brain tissues were homogenized on ice using immunoprecipitation buffer (10 mm Tris-HCl, pH 7.4, 150 mm NaCl, 2 mm EDTA, and 0.5% Nonidet P-40) plus protease inhibitors (1 *μ*g/mL aprotinin, 1 *μ*g/mL leupeptin, and 1 *μ*g/mL pepstatin A). The lysates were collected, centrifuged at 12,000 rpm for 10 min, and quantified for total protein with the bicinchoninic acid protein assay kit (Pierce Technology Co., Iselin, NJ).

### 2.5. Western Blotting

Frozen mouse brain tissues and hippocampal tissues were homogenized, and the lysates were prepared in ice-cold lysis buffer. Total proteins were collected and normalized to equal amounts, as measured by the bicinchoninic acid method. Seventy micrograms of protein from each sample was separated on a sodium dodecyl sulfate-polyacrylamide gel and transferred to polyvinylidene fluoride membranes. The membranes were blocked with 5% nonfat milk and incubated overnight with the primary anti-PPAR-*γ* antibody (1 : 1000; ab8934, Abcam, Cambridge, MA, USA) and anti-*β*-actin antibody (1 : 5000; ab156302, Abcam, Cambridge, MA, USA) at 4°C, followed by incubation with the suitable HRP-conjugated secondary antibody for 4 h. Beta-actin protein was immunodetected as the internal standard. Quantification of Western blot was determined by the ratio of PPAR-*γ* to beta-actin.

### 2.6. BrdU Immunohistochemistry

For the detection of newborn cells in the hippocampus, BrdU-specific immunohistochemistry was performed with the BrdU Immunohistochemistry Kit (ab125306, Abcam, Cambridge, MA, USA). Mice were anesthetized with isoflurane briefly (1.4% isoflurane for 5 min) and perfused transcardially with heparinized saline followed by 4% paraformaldehyde in 0.1 m phosphate buffer at pH 7.4. Mouse brain tissues were removed and kept at 4°C in paraformaldehyde. Serial coronal sections (10 *μ*m) were cut on a cryostat (CM3050 S, Leica Biosystems, Germany) and mounted on coverslips. Each mouse brain was cut for about 30–40 slices. Three hippocampal slices per mouse were examined to reach an average value of CA3 region. Brain sections were deparaffinized and incubated with the quenching solution for 10 min. Two drops of trypsin enzyme were then added to each slide and incubated at room temperature for 10 min, followed by a 3 min rinse in distilled water. Two drops of the denaturing solution were added to each slide and incubated at room temperature for 30 min. The sections were then incubated with blocking buffer at room temperature for 10 min, BrdU antibody (ab6362; 1 : 200, Abcam) at room temperature for 60 min, and streptavidin-horseradish peroxidase (HRP) conjugate at room temperature for 10 min. The integrated optical density (IOD) of nuclei was measured as an approximation of nuclear DNA content with Image-Pro Plus 6.0 software. The IOD among groups was compared by two-way ANOVA test.

### 2.7. Gene Set Enrichment Analysis

Microarray data were obtained from publicly available Gene Expression Omnibus databases (accession number GSE4386). To investigate signaling pathways that were dysregulated in sevoflurane-treated tissues, GSEA was performed, following the developer's protocol (http://www.broad.mit.edu/gsea/). Gene sets are available from the Molecular Signatures DataBase (MolSigDB, http://www.broad.mit.edu/gsea/.msigdb/msigdb_index.html). Briefly, GSEA assesses an enrichment score that reflects the degree to which a gene set is overrepresented at the top or bottom of the entire ranked list of microarray data. The genes are ranked according to changes in expression. In our study, GSEA was performed using default parameters. The permutation number was set as 1000, and the permutation type was set as “gene-sets.”

The heat-map representation of PPAR*γ* signaling-related genes was performed in R. The expression values of each gene were converted to fold changes relative to the control. Log2-fold changes were then generated by color-coding with red and green for overexpression and underexpression, respectively.

### 2.8. Real-Time PCR

Real-time PCR was carried out with SYBR-Green-based reagents (Invitrogen, express SYBR GreenER) using a CFX96 real-time PCR Detection system (BioRad). The relative quantities of immunoprecipitated DNA fragments were calculated using the comparative CT method. Results were compared to a standard curve generated by serial dilutions of input DNA. Data were derived from three independent amplifications. Error bars represent standard deviations.

Primer sequences used for PCR are as follows:

 AP2(Apetala2): F: 5′-GTTGGTGGTGTTTGCTTTGA-3′, R: 5′-TCCATCGATTTCTTGGCTGT-3′,
 Wnt1: F: 5′-CTGGCACGTTGACTCAGAGA-3′, R: 5′-AAGAGCTGCATAGCCACCAC-3′,
 IGF-1: F: 5′-CACCTCTTCTACCTGGCGCT-3′, R: 5′-CGGATAGAGCGGGCTGCTTT-3′,
 IGFBP7: F: 5′-GAGAAGGCCATCACCCAGGTCAGC-3′, R: 5′-GGATCCCGATGACCTCACAGCTCAAG-3′,
 IL-6: F: 5′-GCCCTTCAGGAACAGCTATGA-3′, R: 5′-TGTCAACAACATCAGTCCCAAGA-3′,
 TGF-*α*: F: 5′-ATGAGCACAGAAAGCATGATC-3′, R: 5′-TACAGGCTTGTCACTCGAATT-3′,
 GAPDH: F: 5′-TGGCCTCCAAGGAGTAAGAA-3′, R: 5′-GGTCTGGGATGGAAATTGTG-3′.

### 2.9. Statistical Analysis

Data regarding biochemical changes were expressed as mean ± standard deviation. Data regarding changes in escape latency were expressed as mean ± standard error of the mean. The data for platform crossing time were not distributed normally and thus are expressed as median and interquartile range. The number of samples varied from 6 to 15, and the samples were distributed normally with the exception of platform crossing time (tested by normality test). Interaction between time and group factors in a two-way ANOVA with repeated measurements was used to analyze the difference of learning curves (based on escape latency) between mice in the control group and mice treated with anesthesia in the MWM. The post hoc Bonferroni test was used to compare the difference in escape latency between the control and anesthesia groups in each day of the MWM. The Mann-Whitney *U* test was used to determine the difference between the sevoflurane and control conditions on platform crossing times. There were no missing data for the variables of MWM (escape latency and platform crossing time) during the data analysis. Finally, a two-way ANOVA followed by post hoc Bonferroni test was used to determine differences in the levels of PPAR, TUNEL-positive cells, and BrdU-positive cells among groups. Values of *P* < 0.05 were considered statistically significant. GraphPad software Prism 5 (San Diego CA, USA) was used to analyze the data.

## 3. Results

### 3.1. Multiple Exposures of Sevoflurane-Induced Cognitive Impairment in Young Mice

Children who are frequently exposed to general anesthesia and surgery at an early age may develop impairments in learning and memory [1]. In our previous studies [7], we successfully established an animal model to simulate the effects of multiple exposures of sevoflurane, and this model has allowed us to study the effect of sevoflurane on developmental neurotoxicity.

**Figure 1:**
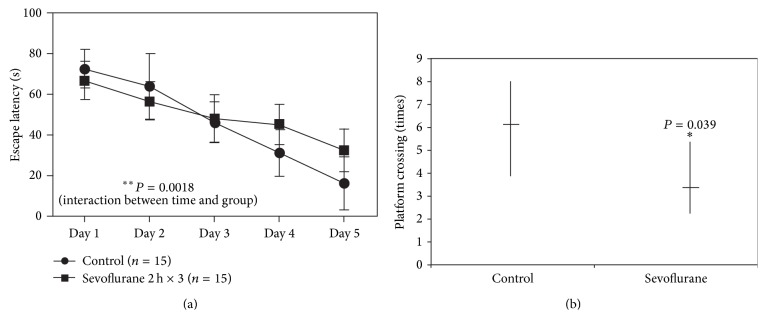
Sevoflurane-induced cognitive impairment in 6-day-old mice. (a) Mice that were exposed to 3% sevoflurane for 2 h daily from postnatal day 6 (P6) to P8 exhibited increased escape latency at P30–P34 in the Morris water maze (MWM), compared with control mice (control: *n* = 15; sevoflurane: *n* = 15). Results from two-way analysis of variance (ANOVA) with repeated measurement analysis showed that there was a statistically significant interaction between time and group, based on the escape latency of the MWM. The post hoc Bonferroni test showed that the mice that received sevoflurane anesthesia had longer escape latency than the mice following the control condition on P33 and P34. (b) Platform crossing times were reduced in mice (P34) that were exposed to 3% sevoflurane for 2 h daily from P6 to P8 (control: *n* = 15; sevoflurane: *n* = 15).

Mice were treated daily with 3% sevoflurane for 2 h from postnatal day 6 (P6) to P8, after which they were tested in the Morris Water Maze (MWM) from P30 to P34. A comparison of the time that each mouse took to reach the platform during reference training (escape latency) showed that there was a statistically significant interaction between time and group (Figure 1(a)) (*P* = 0.0018, two-way ANOVA with repeated measurement). The post hoc Bonferroni test showed that the mice that received sevoflurane anesthesia had longer escape latency than the mice following the control condition on P33 and P34. Furthermore, sevoflurane-treated mice (*n* = 15) had decreased platform crossing times (median, 3; interquartile range, 3–5.5), which represented the number of times that each mouse had crossed the location of the absent platform at the end of reference training, as compared to control mice (*n* = 15) (median, 6; interquartile range 6–8) (Figure 1(b)) (*P* = 0.039, Mann-Whitney *U* test). There was no significant difference in mouse swimming speed between the mice in the sevoflurane anesthesia group and the mice in the control group (data not shown). These data suggested that multiple sevoflurane exposures in young mice might induce cognitive impairment after 3 weeks.

### 3.2. Sevoflurane Induced Neuronal Apoptosis, Neurogenesis Inhibition, and Learning and Memory Impairment through the PPAR-*γ* Signaling Pathway in 6-Day-Old Mice

**Figure 2:**
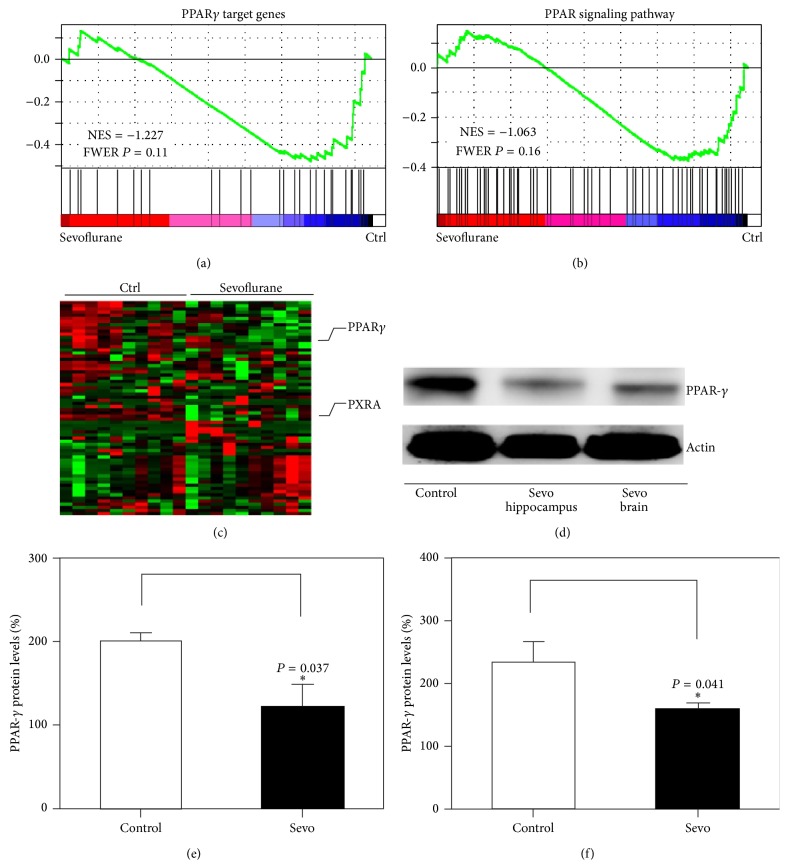
Sevoflurane downregulated PPAR-*γ* expression. (a-b) Gene Set Enrichment Analysis plots for the peroxisome proliferator-activated receptor- (PPAR-) *γ* target gene set and PPAR signaling gene set in the microarray dataset are shown. The PPAR-*γ* target gene signature and PPAR signaling pathway signature were highly enriched in control samples than in sevoflurane-treated samples, thus indicating that PPAR-*γ* signaling is downregulated by sevoflurane. (c) The heat map of PPAR-*γ* target genes in control and sevoflurane-treated samples is shown. (d) Exposure to 3% sevoflurane for 2 h daily from P6 to P8 downregulated the expression of PPAR-*γ* in the brain tissue of mice and the hippocampal tissues of mice. (e) Quantification of western blots (*n* = 6) confirmed that sevoflurane downregulated the expression of PPAR-*γ*. (f) Quantification of western blots (*n* = 6) confirmed that sevoflurane downregulated the expression of PPAR-*γ*. NES: normalized enrichment scores; FWER: familywise error rate; RXRA: Rerinoid X receptor alpha.

**Figure 3:**
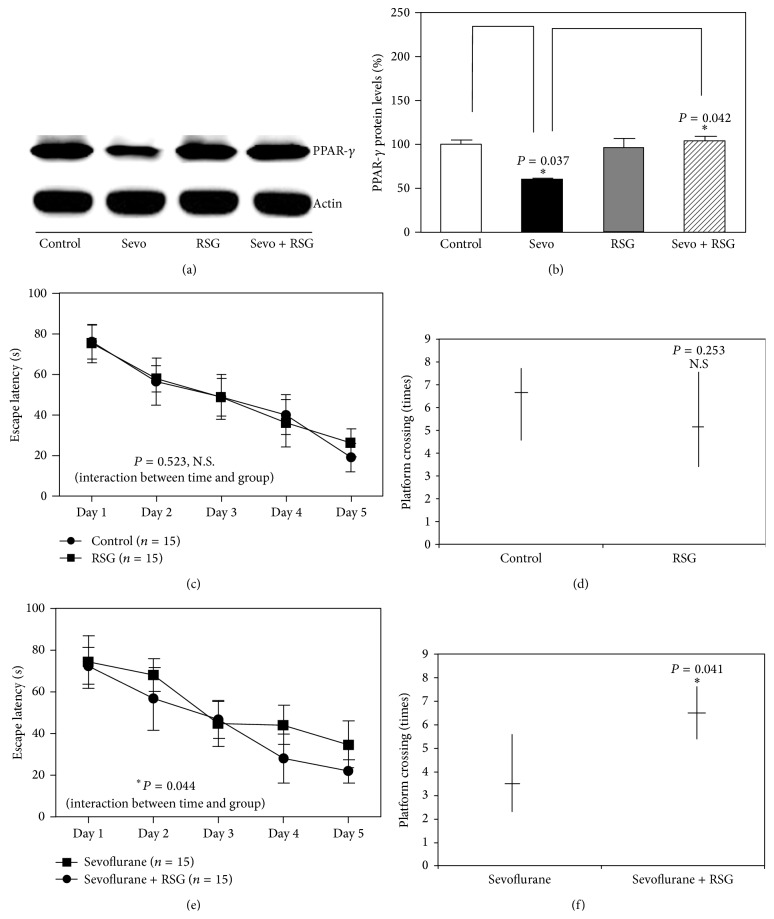
RSG attenuated sevoflurane-induced cognitive impairment in 6-day-old mice. (a) Sevoflurane downregulated the protein levels of PPAR-*γ*, and rosiglitazone (RSG) attenuated this decrease in PPAR-*γ* levels in 6-day-old mice. (b) Quantification of western blots confirmed that RSG attenuated the sevoflurane-induced decrease in the protein levels of PPAR-*γ* (*n* = 6). (c) Daily treatment of P6 mice with RSG for 3 days did not increase the escape latency of mice in the MWM at P30–P34 (control: *n* = 15; RSG: *n* = 15). (d) RSG treatment did not reduce the platform crossing times of mice at P34 (control: *n* = 15; RSG: *n* = 15). (e) Results from two-way ANOVA with repeated measurement analysis showed that there was a statistically significant interaction between time and group (sevoflurane: *n* = 15; sevoflurane + RSG: *n* = 15). (f) The Mann-Whitney test showed that there was a significant difference in platform crossing times between sevoflurane-treated mice and sevoflurane + RSG-treated mice (sevoflurane: *n* = 15; sevoflurane + RSG: *n* = 15).

**Figure 4:**
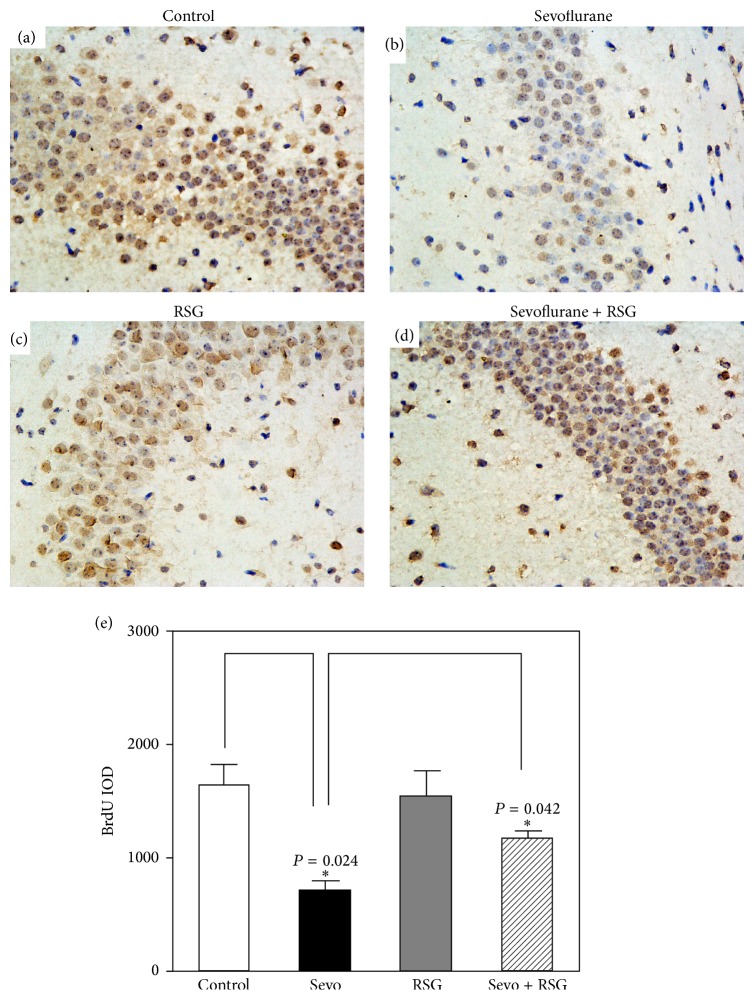
Sevoflurane-induced neuronal apoptosis and neurogenesis inhibition through the PPAR*γ* signaling pathway in 6-day-old mice. (a-b) Exposure of P6 mice to sevoflurane for 2 h daily for 3 days induced neurogenesis inhibition. (c-d) RSG attenuated sevoflurane-induced neurogenesis inhibition. (e) There was a statistically significant difference in the number of 5-bromodeoxyuridine- (BrdU-) positive cells between sevoflurane-treated mice and sevoflurane + RSG-treated mice (two-way ANOVA; sevoflurane: *n* = 9; sevoflurane + RSG: *n* = 9). IOD: integrated optical density.

Sevoflurane has been shown to inhibit neurogenesis, induce neuronal apoptosis, and impair learning and memory [3, 7]. However, the mechanism underlying this effect is still unknown. Given the findings that PPAR-*γ* is essential for cell survival and cognitive function [13]. We therefore assessed the effects of sevoflurane on PPAR-*γ* target genes, as well as PPAR-*γ* expression in brain tissues of the mice. The brain tissues were harvested at the end of sevoflurane anesthesia (P8). To identify signaling pathways that contribute to sevoflurane's effects, we conducted gene set enrichment analysis (GSEA) using the KEGG suite and Reactome suite on microarray data obtained from the publicly available GEO databases (accession number GSE4386). Several signaling pathways are differentially regulated between control and sevoflurane treated samples. We selected the PPAR signaling pathway for further validation based on the following criteria: (1) PPAR signaling showed high enrichment in both KEGG gene sets and Reactome gene sets; (2) PPAR pathway has been shown to be involved in neural development and neurodegenerative diseases. In this study, Gene Set Enrichment Analysis (GSEA) plots (Figures 2(a) and 2(b)) and the heat map of PPAR-*γ* target genes (Figure 2(c)) in sevoflurane-treated samples suggested that the PPAR-*γ* signaling pathway may play an important role in sevoflurane-induced neurotoxicity. Indeed, in the hippocampal tissues and the whole brain tissues, sevoflurane downregulated the expression of PPAR-*γ* as compared to the control condition (Figure 2(d)). The quantification of the western blot illustrated that sevoflurane decreased PPAR-*γ* expression in the hippocampal tissues of mice (198 ± 27% versus 126 ± 43%, *n* = 6, *P* = 0.037, two-way ANOVA) (Figure 2(e)). The quantification of the western blot illustrated that sevoflurane decreased PPAR-*γ* expression in the brain tissue of mice (223 ± 25% versus 178 ± 23%, *n* = 6, *P* = 0.041, two-way ANOVA) (Figure 2(f)). In this experiment, we found similar western blotting results in whole brain and hippocampal tissues after sevoflurane treatment. Because the hippocampal tissues of six-day-old mice were very tiny, we chose to perform western blotting with whole brain tissues in the next experiment. Therefore, the contribution of the PPAR-*γ* signaling pathway to sevoflurane-induced neuronal apoptosis, neurogenesis inhibition, and learning and memory impairment was investigated. RSG is an insulin sensitizer that is a member of the thiazolidinedione class of drugs and binds to PPARs. Specifically, it is a selective ligand of PPAR-*γ*. Because it is well tolerated by humans with few side effects, we used RSG in this study. RSG reversed the sevoflurane-induced decrease in PPAR-*γ* expression (Figure 3(a)) and the quantification of the western blot illustrated that RSG reversed PPAR-*γ* expression when compared to the sevoflurane group (112 ± 17% versus 63 ± 8%, *n* = 6, *P* = 0.042, two-way ANOVA) (Figure 3(b)). MWM study showed that RSG only neither increased escape latency nor decreased platform crossing times when compared with the control group (Figures 3(c) and 3(d)). Results from two-way analysis of variance (ANOVA) with repeated measurement analysis showed that the interaction between time and group, based on the escape latency of the MWM, was statistically significant between sevoflurane-treated mice (*n* = 15) and sevoflurane + RSG-treated mice (*n* = 15) (*P* = 0.044, two-way ANOVA with repeated measurement). The post hoc Bonferroni test shows that the mice that received sevoflurane anesthesia had longer escape latency than the sevoflurane + RSG-treated mice on P33 (Figure 3(e)). Sevoflurane-treated mice had decreased platform crossing times (*n* = 15) (median, 3.5; interquartile range, 3.5–5) as compared to sevofluran + RSG group mice (*n* = 15) (median, 6.5; interquartile range 6.5–8) (*P* = 0.041, Mann-Whitney *U* test) (Figure 3(f)). Immunohistochemistry images showed the density of 5-bromo-2-deoxyuridine- (BrdU-) positive cells in hippocampus of mice (P8) in control condition (*n* = 9), sevoflurane anesthesia (*n* = 9), RSG (*n* = 9), and RSG plus sevoflurane anesthesia (*n* = 9), respectively (Figures 4(a)–4(d)). The IOD of the sevoflurane group was much smaller than those of the control group and RSG + sevoflurane group (645 ± 54% versus 1737 ± 87%, *P* = 0.0024; 645 ± 54% versus 1129 ± 72%, *P* = 0.042, two-way ANOVA), which showed that sevoflurane inhibited neurogenesis, but RSR mitigated sevoflurane-induced inhibition of neurogenesis in hippocampus (Figure 4(e)). These results suggested that sevoflurane induced neurogenesis inhibition, and learning and memory impairment through the PPAR-*γ* signaling pathway.

### 3.3. EE Attenuated Sevoflurane-Induced Neuronal Apoptosis, Neurogenesis Inhibition, and Learning and Memory Impairment through the PPAR*γ* Signaling Pathway in 6-Day-Old Mice

**Figure 5:**
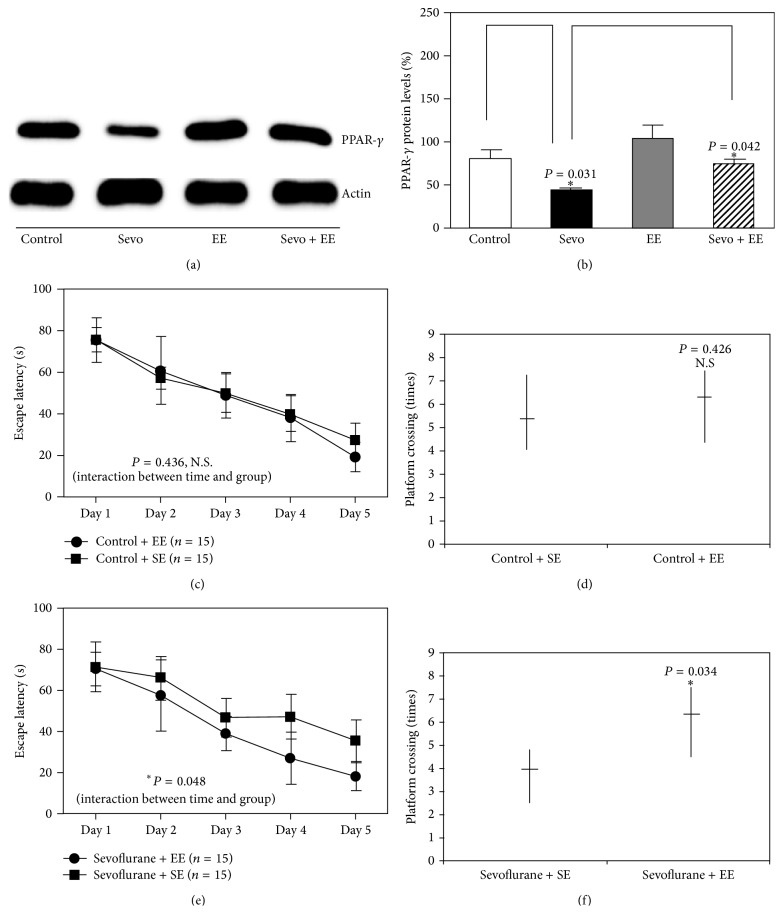
EE attenuated sevoflurane-induced cognitive impairment in 6-day-old mice. (a) Sevoflurane downregulated the protein levels of PPAR-*γ*, and exposure to environmental enrichment (EE) for 2 h daily from P8 to P30 attenuated this decrease in PPAR-*γ* levels. (b) Quantification of western blots confirmed that EE attenuated the sevoflurane-induced decrease in the protein levels of PPAR-*γ* (*n* = 6). (c) EE did not increase the escape latency of mice in the MWM, as compared to the control condition (tested from P30 to P34) (control, *n* = 15; EE, *n* = 15). (d) EE had no effect on the platform crossing times of mice at P34, as compared to the standard environment (SE) (control: *n* = 15; EE: *n* = 15). (e) There was a statistically significant interaction of time and group, based on the escape latency of the MWM, between mice that were exposed to sevoflurane and mice that were exposed to both sevoflurane and EE (two-way ANOVA; sevoflurane: *n* = 15; sevoflurane + EE: *n* = 15). (f) The Mann-Whitney test showed that there was a significant difference in the platform crossing times between mice that were exposed to sevoflurane and mice that were exposed to both sevoflurane and EE (sevoflurane: *n* = 15; sevoflurane + EE: *n* = 15).

We have previously shown that EE can attenuate sevoflurane-induced learning and memory impairment [7]. However, the mechanism by which this occurs is unclear. Next, we investigated whether EE can mitigate sevoflurane-related neurotoxicity through the PPAR*γ* signaling pathway. We tested the mice by MWM from P30 to P34. We harvested the brain tissues at P30. EE attenuated the sevoflurane-induced decrease in PPAR*γ* expression (Figure 5(a)). The quantification of the western blot illustrated that EE attenuated sevoflurane-induced decrease in PPAR*γ* expression when compared to sevoflurane group (76 ± 17% versus 44 ± 9%, *n* = 6, *P* = 0.042, two-way ANOVA) (Figure 5(b)). MWM study showed that both escape latency and platform crossing times were not statistically significant between the mice in standard condition (*n* = 15) and environmental enrichment (*n* = 15) (Figures 5(c) and 5(d)). The interaction between time and group, based on the escape latency of the MWM, was statistically significant between the sevoflurane and EE groups, as demonstrated by two-way ANOVA with repeated measurement analysis (Figure 5(e)) (*P* = 0.048, two-way ANOVA with repeated measurement). The post hoc Bonferroni test shows that the mice that received sevoflurane anesthesia had longer escape latency than the mice following the control condition on P33 and P34. Furthermore, the platform crossing times between sevoflurane (*n* = 15) (median, 4; interquartile range, 4–6) and EE groups (*n* = 15) (median, 6.3; interquartile range, 6.3–7) were significantly different (Figure 5(f)) (*P* = 0.034, Mann-Whitney test).

**Figure 6:**
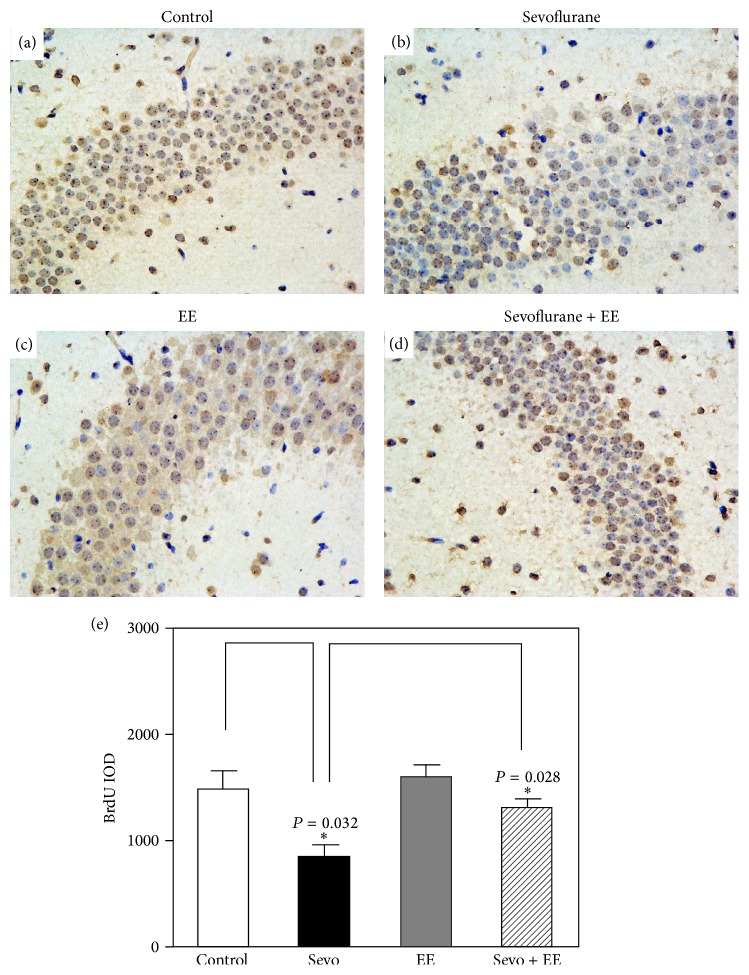
EE attenuated sevoflurane-induced neuronal apoptosis and neurogenesis inhibition through the PPAR*γ* signaling pathway. (a-b) Multiple sevoflurane exposures from P6 to P8 induced neurogenesis inhibition in young mice (P30). (c-d) EE attenuated sevoflurane-induced neurogenesis inhibition. (e) The number of BrdU-positive cells was significantly different between mice that were exposed to sevoflurane and mice that were exposed to both sevoflurane and EE (two-way ANOVA; sevoflurane: *n* = 9; sevoflurane + EE: *n* = 9).

Immunohistochemistry images showed the density of 5-bromo-2-deoxyuridine- (BrdU-) positive cells in hippocampus of mice (P30) in control condition (*n* = 9), sevoflurane anesthesia (*n* = 9), RSG (*n* = 9), and RSG plus sevoflurane anesthesia (*n* = 9), respectively (Figures 6(a)–6(d)). The IOD of the sevoflurane group was much smaller than those of the control group (712 ± 53% versus 1447 ± 103%, *n* = 9, *P* = 0.032). Moreover, the IOD of the sevoflurane group was also much smaller than those of the EE + sevoflurane group (712 ± 53% versus 1257 ± 83%, *n* = 9, *P* = 0.028). These results showed that sevoflurane inhibited neurogenesis, but EE mitigated sevoflurane-induced inhibition of neurogenesis in hippocampus (Figure 6(e)). These results suggested that EE can attenuate the effects of sevoflurane on neuronal apoptosis, neurogenesis inhibition, and learning and memory impairment through the PPAR*γ* signaling pathway.

## 4. Discussion

Sevoflurane is the most widely used anesthetic in clinical practice. In young mice, it has been shown to induce apoptosis, inhibit neurogenesis, and cause learning and memory impairment [7]. Children who are frequently exposed to general anesthesia and surgery at an early age may develop learning and memory impairment. Potential neurotoxicity in the developing brain has engendered considerable concern by the U.S. FDA [2]. We have previously shown that EE can rescue sevoflurane-induced learning and memory impairment in young mice. This finding may pave the way for the development of a therapeutic approach to prevent or treat sevoflurane-induced learning and memory impairment. The purpose of this study was to explore the mechanism of EE.

The hippocampus is known to be important for learning and memory function, and new neurons in the hippocampus of rodents and humans are assumed to be important for maintaining memory function [11, 14, 18]. Some studies have shown that sevoflurane not only induces neuronal apoptosis but also inhibits neurogenesis. In the current study, multiple exposures of sevoflurane increased neuronal apoptosis and decreased the number of newborn neurons in the hippocampus of 6-day-old mice. These findings are consistent with other studies and suggest that sevoflurane not only induces apoptosis but also inhibits neurogenesis in the hippocampus of young mice [1]. EE, which is defined as a combination of “complex inanimate objects and social stimulation,” has been shown to improve learning and memory function by increasing hippocampal neurons, improving spatial abilities, and enhancing dendritic growth, thereby increasing neurogenesis [16, 18–20]. Interestingly, our previous studies showed that EE can rescue sevoflurane-induced learning and memory impairment. In this study, EE mitigated sevoflurane-induced neuronal apoptosis and neurogenesis inhibition. This suggests that targeting apoptosis/neurogenesis may potentially prevent or treat sevoflurane-induced learning and memory impairment.

In this study, we demonstrate that PPAR-*γ* expression was downregulated after sevoflurane exposure. PPAR-*γ* is a key transcription factor that has neuroprotective effects, including cell survival and cognitive enhancement, in disease-related behavioral impairment. Moreover, RSG is a PPAR-*γ* agonist that has been shown to attenuate the effects of sevoflurane on neuronal apoptosis, neurogenesis inhibition, and learning and memory impairment [17, 21]. These findings suggest that, at least in part, sevoflurane induces neurotoxicity by downregulating PPAR-*γ*. In addition, EE rescued sevoflurane-induced neurotoxicity and learning and memory impairment by increasing PPAR-*γ* levels in the hippocampus. This implies that the PPAR-*γ* signaling pathway may also be a potential therapeutic target for preventing or treating sevoflurane-induced neurotoxicity.

This study has several limitations. First, PPAR-*γ* has been shown to exhibit anti-inflammatory effects and enhance synaptic function. In previous studies, we found that sevoflurane induces neurotoxicity and learning and memory impairment by increasing neuroinflammation and inhibiting synaptic function [7]. In the current study, we only measured neuronal apoptosis and neurogenesis. Thus, further studies are needed to test this hypothesis. Second, we treated the mice with 3% sevoflurane rather than the gradient dose. Three percent sevoflurane is a clinically relevant concentration that has been shown to induce apoptosis in H4 human neuroglioma cells and neurons. Furthermore, it can increase learning and memory disability [5, 12].

## Supplementary Material

To explore whether the PPARγ target genes are affected by sevoflurane, we test several PPARγ target genes such like AP2,Wnt1, IGF-1,IGFBP7, Il-6 and TNF-α by RT-PCR. Finally, we found that these several PPARγ target genes were dramatically increased at mRNA levels (Supply figure A).Moreover, in order to support their conclusion further, we test if PPARγ antagonist abolishes the effect of environmental enrichment on sevoflurane treatment. The mice were treated with PPARγ antagonist GW 6471(5mg/kg) intraperitoneally before it was put in the EE every day for 2 h from P8–P30. At the end, we found that PPARγ antagonist abolishes the effect of environmental enrichment on Sevoflurane treatment (Supply figure B).
