# Supplementary figures and images for: Environmental Enrichment Attenuated Sevoflurane-Induced Neurotoxicity through the PPAR-γ Signaling Pathway
Source: Biomed Res Int. 2015 Jul 6;2015:107149. doi: 10.1155/2015/107149 (PMC4506847; doi:10.1155/2015/107149)

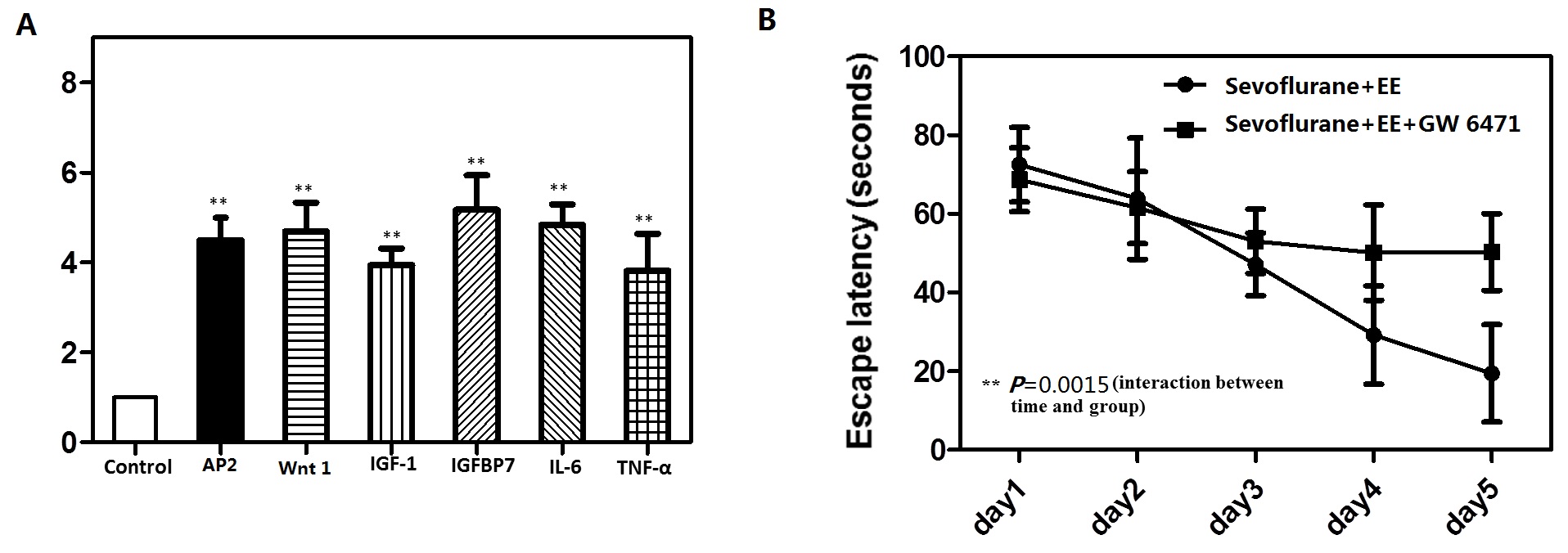

Supplement: Supplementary file 1 — To explore whether the PPARγ target genes are affected by sevoflurane, we test several PPARγ target genes such like AP2,Wnt1, IGF-1,IGFBP7, Il-6 and TNF-α by RT-PCR. Finally, we found that these several PPARγ target genes were dramatically increased at mRNA levels (Supply figure A).Moreover, in order to support their conclusion further, we test if PPARγ antagonist abolishes the effect of environmental enrichment on sevoflurane treatment. The mice were treated with PPARγ antagonist GW 6471(5mg/kg) intraperitoneally before it was put in the EE every day for 2 h from P8–P30. At the end, we found that PPARγ antagonist abolishes the effect of environmental enrichment on Sevoflurane treatment (Supply figure B). [file 107149.f1.jpg]
